# The role of virtual reality-based cognitive training in enhancing motivation and cognitive functions in individuals with chronic stroke

**DOI:** 10.1038/s41598-025-08173-1

**Published:** 2025-07-12

**Authors:** Maria Grazia Maggio, Lilla Bonanno, Amelia Rizzo, Martina Barbera, Alessandra Benenati, Federica Impellizzeri, Francesco Corallo, Rosaria De Luca, Angelo Quartarone, Rocco Salvatore Calabrò

**Affiliations:** 1 IRCCS Centro Neurolesi Bonino Pulejo, 98124 Messina, Italy; 2 University of Messina, Piazza Pugliatti, 1, 98122 Messina, Italy; 3 Department of Psychology “Renzo Canestrari”, University of Bologna, Bologna, Italy

**Keywords:** Motivation, Psychology

## Abstract

Stroke represents a major health challenge worldwide, often resulting in significant long-term disability that affects cognitive, motor, and emotional functions. Rehabilitation strategies that enhance patient motivation are crucial for improving outcomes. This randomized controlled trial investigated the impact of Virtual Reality Rehabilitation Systems (VRRS) compared to traditional cognitive training on motivation, cognitive recovery, and emotional state in post-stroke patients. Fifty-four adults with chronic stroke were randomized into two equal groups (27 participants per group). The experimental group received 24 sessions of Virtual Reality (VR) cognitive training, while the control group underwent 24 sessions of traditional cognitive rehabilitation. Motivation was assessed using the McClelland test, while cognitive and emotional states were evaluated using the Montreal Cognitive Assessment (MoCA) and Hamilton Rating Scales for Anxiety and Depression (HAM-A, HAM-D). The experimental group exhibited significant improvements in motivation, with marked increases in Achievement (T0: 68.41 ± 15.81, T1: 68.93 ± 15.80; p < 0.001) and Affiliation(T0: 60.67 ± 14.64, T1: 60.93 ± 15.59; p = 0.006) dimensions, alongside enhanced cognitive function (T0: 24.781 ± 1.89, T1: 26 (24.5–27); p = 0.001), reduced depressive (T0: 41 ± 2.32, T1: 6 (4–8); p = 0.003) and anxiety symptoms (T0: 4.26 ± 1.99, T1: 3.30 ± 1.94; p < 0.001). The Control Group showed significant differences only in MOCA (T0: 25 (23–26.5), T1: 25 (24–27); p < 0.001). Between-group analysis revealed no significant differences between the two groups. These findings underscore the potential of VR as a multifaceted tool to boost motivation, facilitate cognitive recovery, and improve emotional state, offering a comprehensive approach to post-stroke rehabilitation.

## Introduction

Stroke is a principal cause of morbidity and mortality worldwide. It has an estimated incidence of 15 million new cases each year, of which approximately 5 million leads to permanent disability^1^. In Italy, approximately 200,000 people suffer from stroke each year, reducing quality of life for survivors and their families^2^. The neurological consequences of stroke can manifest in various ways, including motor, cognitive, and emotional difficulties, with a severe impact on the autonomy and general well-being of patients. Cognitive dysfunctions could include deficits in attention, executive dysfunction, memory, and reasoning ability^3^, increasing the complexity of recovery and requiring a multidisciplinary and personalized rehabilitation approach. Notably, the chronic phase of stroke recovery, defined as the phase six months or more after the event, requires special attention for the difficulty faced by the patients^4,5^. Unlike the subacute phase, during which spontaneous biological recovery often occurs, in the chronic phase, the neurological deficits can be largely stabilized^4,6^. In this phase, individuals often experience persistent cognitive deficits that significantly interfere with daily functioning and independence.

Moreover, the prolonged time since the initial event is often associated with increased emotional symptoms, including ongoing depression, anxiety, and apathy, which can seriously impact engagement in rehabilitation^7,8^. The loss of motivation is a common challenge in the long-term phase of the rehabilitation process. Individuals could feel their progress stalled, resulting in hopelessness or resignation. The repetitive and often tedious nature of traditional long-term therapy may further contribute to treatment fatigue and poor adherence^9,10^. Therefore, people living with chronic stroke represent a population with specific needs for innovative treatment approaches. These interventions can effectively address persistent cognitive and emotional difficulties and help restore and sustain motivation for prolonged rehabilitation^11^. Post-stroke rehabilitation is essential, and it should adopt a patient-centered approach to support the recovery process^12^. Several studies have shown that active rehabilitation approaches, where the patient is involved in the rehabilitation process, are more effective in improving functional and cognitive function than passive approaches^13–15^. In this perspective, the increasing integration of innovative technologies, such as virtual reality (VR), has transformed the rehabilitation landscape by offering immersive experiences that stimulate not only motor skills, but also cognitive functions and, above all, patient motivation. In rehabilitation, VR provides patients with a safe, controlled, and engaging environment in which they can train their skills and experience success with awareness of their performance and the outcome of their movement^16–19^. This approach helps reduce anxiety and improves therapeutic engagement^20,21^ VR systems offer unique features, such as motion tracking, augmented feedback, and sensor integration, that make them particularly suitable for rehabilitation and integration into clinical practice. In particular, VR creates immersive and interactive environments, which allow the subject to feel immersed in a virtual environment. This sense of “presence” can increase engagement, making therapy less clinical and more intrinsically motivating, potentially shifting attention from the effort involved to the task being performed^22–24^. VR can supports structured motor learning and cognitive training^25^. Indeed, elements such as points, levels, real-time feedback, and narrative goals can stimulate both extrinsic and intrinsic motivation, making repetitive practice more enjoyable and rewarding^26^. Furthermore, VR allows for precise control over task parameters, enabling therapists to tailor the difficulty of cognitive and motor exercises to match each patient’s abilities^16^. This personalization helps ensure that tasks remain challenging yet achievable, reducing frustration and fostering self-efficacy, an essential component for maintaining long-term motivation^27^. In VR settings, feedback is immediate and multisensory, visual, auditory, allowing patients to quickly understand the consequences of their actions^19,28^. This immediacy helps correct errors, reinforce effective strategies, and facilitate in traditional rehabilitation^29^. Importantly, VR tasks can be specifically designed to target a wide range of cognitive functions commonly affected by stroke^17^. For example, navigating complex virtual environments can improve visuospatial abilities and planning; simulated daily-life tasks like shopping or cooking can engage executive functions such as sequencing, problem-solving, and working memory; and attention-based games can help train sustained focus and resistance to distraction^30^. The immersive, multisensory, and cognitively demanding nature of these exercises is believed to support neuroplastic changes that underpin functional recovery^31^. VR provides a safe space for patients to rehearse difficult tasks, such as crossing a virtual street or managing social interactions, without real-world risks or the anxiety they often provoke. This can help build confidence and reduce avoidance behaviors tied to perceived limitations^20^. Taken together, the combination of enhanced engagement, individualized challenge, immediate feedback, targeted cognitive stimulation, and safe practice environments provides a strong scientific foundation for exploring VR as a tool to improve emotional, cognitive rehabilitation, and motivation symptoms in stroke survivors. In particular, motivation is the level of energy, commitment, and perseverance a patient demonstrates in pursuing specific goals during therapy. It plays a critical role in the rehabilitation process^32^. Its importance cannot be overstated, as rehabilitation is inherently a challenging, often lengthy, and sometimes frustrating process and requires constant effort from the patient. Higher levels of motivation are directly associated with greater engagement in therapy. Furthermore, motivation may be linked to improved rehabilitation outcomes, including functional improvements and goal achievement^33^. In addition to its direct behavioral effects, motivation plays a crucial role in enhancing the subjective experience of rehabilitation^33^. Furthermore, it may influence how patients perceive pain or discomfort during exercises^34^. Finally, motivation can act to enhance self-efficacy through the successful completion of challenging tasks and ultimately increase satisfaction with both the therapeutic process and its outcomes^34^. It is therefore essential to understand motivational processes during the rehabilitation process. Self-determination theory (SDT) offers a valuable framework to interpret these processes, highlighting the role of intrinsic and extrinsic motivation. Furthermore, SDT also considers the satisfaction of basic psychological needs, autonomy, competence, and relatedness, as key to sustaining long-term commitment^35^. However, SDT does not fully explain the deeper, often unconscious, motivations that drive behavior over time. To capture a broader motivational profile, our study integrates SDT with McClelland’s needs theory, adopting a dual-theoretical perspective. While SDT informs the interpretation of motivational models, McClelland’s framework provides the basis for assessing patients’ motivational styles, focusing on learned social needs: Achievement (ACH), Affiliation (AFF), and Power (POW)^36^. These needs, often implicit and enduring, represent internal forces that energize and drive behavior, particularly in goal-oriented contexts such as rehabilitation. Although conceptually distinct, McClelland’s theory complements SDT by illuminating underlying motivational changes, such as a transition from power-oriented to achievement-oriented motivation, which may reflect a movement toward more autonomous and self-determined engagement^37^. Although motivational profiles are considered crucial in rehabilitation, their specific impact on cognitive and emotional functioning, particularly in the context of VR-based interventions, remains largely unexplored. To address this, we conducted a randomized controlled trial (RCT) comparing the effects of VR-based cognitive rehabilitation to conventional cognitive training in individuals with chronic stroke. Specifically, the study had three main objectives:

- (1) to evaluate changes in motivation as the primary outcome,
- (2) to evaluate cognitive and emotional changes as secondary outcomes, and
- (3) to explore whether changes in motivation were associated with changes in cognitive and emotional functioning. emotional state

## Methods

### Study design and population

This study was a single-blind randomized controlled trial (RCT), in which outcome assessors were blinded to group allocation. This RCT study included fifty-four patients diagnosed with stroke (either hemorrhagic or ischemic) who attended the Robotic and Behavioral Neurorehabilitation Unit of the IRCCS Centro Neurolesi "Bonino-Pulejo" in Messina, Italy, between October 2023 and December 2024.

The sample size was determined based on an a priori power analysis using G*Power 3.1.9.7 software. Considering a medium-to-large effect size (Cohen’s d = 0.80), a significance level of 0.05, and a power of 0.80, the required sample size was calculated to be 52 participants in total, with a minimum of 26 participants per group. To account for potential dropouts or incomplete data, 27 participants per group were included, resulting in a total of 54 participants.

All patients were randomly assigned to one of two groups using block randomization to ensure an equal number of participants per group and minimize potential biases over time. Blocks of four participants were created, and within each block, patients were randomly allocated to either the control group or the experimental group. The Control group included 27 patients who received traditional cognitive training, and the Experimental Group included 27 patients who underwent training using the Virtual Reality Rehabilitation System (VRRS).

Participants were included in the study if they met the following criteria:

i) Clinical and neuroradiological diagnosis of ischemic or hemorrhagic stroke in the chronic phase (i.e., at least 6 months post-acute neurological event); ii) Age between 18 and 80 years; iii) Absence of severe cognitive impairment, as indicated by a Montreal Cognitive Assessment (MoCA) score greater than 18, as individuals with severe cognitive impairment were considered unable to comprehend instructions and actively engage in the training, particularly in the VR condition; iv) Ability to follow instructions and participate in rehabilitation activities.

The exclusion criteria were: i) Presence of significant medical comorbidities that could interfere with rehabilitation (e.g., severe cardiac or respiratory disease); ii) Disabling sensory alterations affecting the ability to perform the training (e.g., severe visual or hearing impairment); iii) Severe psychiatric disorders that could affect participation or compliance (e.g., major depression or psychosis); iv) Any neurological conditions other than stroke that could affect cognitive or motor functions (e.g., dementia, Parkinson’s disease);

v) Recent surgery or medical intervention that could limit rehabilitation participation.

To ensure balance between groups, baseline characteristics such as age, type of stroke (ischemic or hemorrhagic), and MoCA scores were recorded and compared. No significant differences were observed, indicating the groups were well-matched. Dropouts were managed using an intention-to-treat approach, ensuring that all randomized participants were included in the final analysis regardless of their level of adherence to the intervention.

### Ethical aspects

This randomized controlled trial (RCT) analyzed data collected as part of a larger multicenter study registered at ClinicalTrials.gov (NCT05703906, registered on 12/12/2022; and updated on 30/01/2023; https://clinicaltrials.gov/study/NCT05703906). The original trial was approved by the Ethics Committee for Clinical Trials of the Province of Venice and IRCCS San Camillo (Coordinator Centre, Prot. 2017.16), alongside all other participating centers. In 2023, a protocol amendment (Prot. 05/2023) was approved by the Ethics Committee at IRCCS Centro Neurolesi Bonino Pulejo to include additional analyses focusing on emotional and motivational outcomes. These analyses were conducted exclusively on patients enrolled at our center.

The study was conducted following the principles of the Declaration of Helsinki. All participants obtained Written informed consent before enrollment, ensuring they were fully informed of their right to withdraw at any time. Confidentiality was maintained by anonymizing data through unique identification codes and securely storing all records.

### Outcome measures

Data collection and analysis were performed by blinded assessors to minimize potential biases. Motivation was evaluated using the McClelland test. a tool designed to identify the predominant need driving an individual’s behaviour. The test evaluates how people respond to different situations and which motivations influence their decisions through three primary subscales: ACH, which measures the drive to excel and succeed in challenging tasks; AFF, which assesses the desire for relationships and social connections; and POW, which reflects the motivation to influence others and exert control over one’s environment.

Cognitive function was measured using the Montreal Cognitive Assessment (MoCA), which assesses various cognitive functions, including attention, memory, and executive function.

Emotional state was assessed with the Hamilton Anxiety Rating Scale (HAM-A) and Hamilton Depression Rating Scale (HAM-D), providing information on levels of anxiety and depression among participants.

A skilled neuropsychologist administered each test or scale at both the beginning and end of the intervention to evaluate cognitive and emotional changes resulting from the training programs. All outcome measures were administered by independent evaluators who were blinded to group allocation and were not involved in the delivery of the interventions, in order to reduce potential bias.

A summary of the outcome measures, including interpretative guidelines and clinical cut-offs where applicable, is provided in Table 1.

**Table 1:** Summary of outcome measures.

Measure	Interpretation of scores	Notes on interpretation	Psychometric properties
Montreal cognitive assessment (MoCA)	Better cognitive functioning	Cut-off: < 26 suggests possible cognitive impairment	Good internal consistency (α = 0.83); test–retest reliability r = 0.92; validated in stroke and neurological populations^38^
Hamilton depression rating scale (HAM-D)	Greater severity of depressive symptoms	Cut-offs: 0–7 = normal; 8–16 = mild; 17–23 = moderate; ≥ 24 = severe	High inter-rater reliability (ICC = 0.82–0.98); Cronbach’s α ≈ 0.79; widely validated in clinical populations^39^
Hamilton anxiety rating scale (HAM-A)	Greater severity of anxiety	Cut-offs: < 17 = mild; 18–24 = moderate; ≥ 25 = severe	Good internal consistency (α = 0.85); test–retest reliability r = 0.86; validated in anxiety-related disorders^40^
McClelland test^41^			
Achievement (ACH)	Greater alignment with achievement motivation	Higher score relative to the other two subscales indicates dominance of Achievement profile	Based on the Thematic Apperception Test (TAT); inter-rater reliability reported up to 95% when using objective coding criteria; construct validity supported by numerous empirical studies
Affiliation (AFF)	Greater alignment with affiliation motivation	Higher score relative to the other two subscales indicates dominance of Affiliation profile	See above; used in motivational style profiling; evidence of predictive validity in behavioral studies (Schultheiss & Brunstein, 2005)
Power (POW)	Greater alignment with power motivation	Higher score relative to the other two subscales indicates dominance of Power profile	See above; reliable differentiation across motivational domains; used in health psychology and behavioral motivation research

### Procedures

All study participants underwent cognitive rehabilitation three times a week for eight weeks, completing a total of 24 sessions, each lasting approximately 45 minutes. These sessions targeted specific cognitive functions, with exercise complexity adjusted based on individual progress. Progress was evaluated through successful responses (e.g., achieving at least 9 out of 10 correct answers) while maintaining a low error rate (fewer than one mistake).

Both groups participated in the same number of neurorehabilitation sessions (Table 2); however, only the experimental group received cognitive training using the Virtual Reality Rehabilitation System (VRRS). The control group engaged in traditional cognitive rehabilitation, which involved completing paper-based worksheets containing various cognitive exercises and games, such as puzzles and memory tasks, aimed at enhancing different cognitive abilities. Control Group participants performed the exercises independently. To reduce learning bias, the type and difficulty of the exercises were systematically and randomly adjusted throughout the study. All interventions were conducted by licensed neuropsychologists with expertise in cognitive rehabilitation. The therapists responsible for providing the training were not involved in outcome assessments. Therapists in the experimental group received specific training in the use of the VRRS system before the beginning of the study, under the supervision of certified personnel. Both VR-based and traditional interventions were delivered in quiet, dedicated rooms within the same clinical unit, under comparable environmental conditions.

**Table 2:** Cognitive rehabilitative program.

Cognitive domain	VRRS cognitive training	Traditional cognitive training
Orientation	Tasks include sorting days and months in order, recognizing object positions (e.g., right, left), and identifying rotation types (clockwise, counterclockwise). Difficulty increases with more elements	Stimulates spatial and temporal orientation through recall of personal and general information (e.g., events, places, dates). Delivered visually, verbally, or auditorily. Tasks grow in specificity and detail over time
Attention	Interactive tasks in a virtual environment where patients select targets (e.g., colors, animals) within a set time. Feedback is provided through audio-visual cues. Difficulty increases with distractors and time constraints	Patients identify and touch target stimuli (e.g., specific colors, animals) while ignoring distractors. Tasks become progressively more complex, adjusting for the type and number of targets and distractors
Memory	Patients observe elements (e.g., colors, objects, numbers) and recall them in a virtual environment. Tasks train visuospatial and verbal memory. Difficulty increases with more elements and shorter recall times	Paper-and-pencil tasks include recalling item sequences, digits, or spatial positions. Conducted face-to-face, the training progresses through three levels of complexity based on time and stimuli
Executive and visuospatial functions	Patients perform goal-directed movements (e.g., touching, manipulating objects) in a virtual environment. Tasks involve ideomotor sequences with increasing complexity and provide immediate feedback	Patients execute movements using physical tools (e.g., pencils) and complete associations (e.g., letter-color) during face-to-face sessions. Complexity increases through three levels of ideomotor sequences

**Fig. 1:**
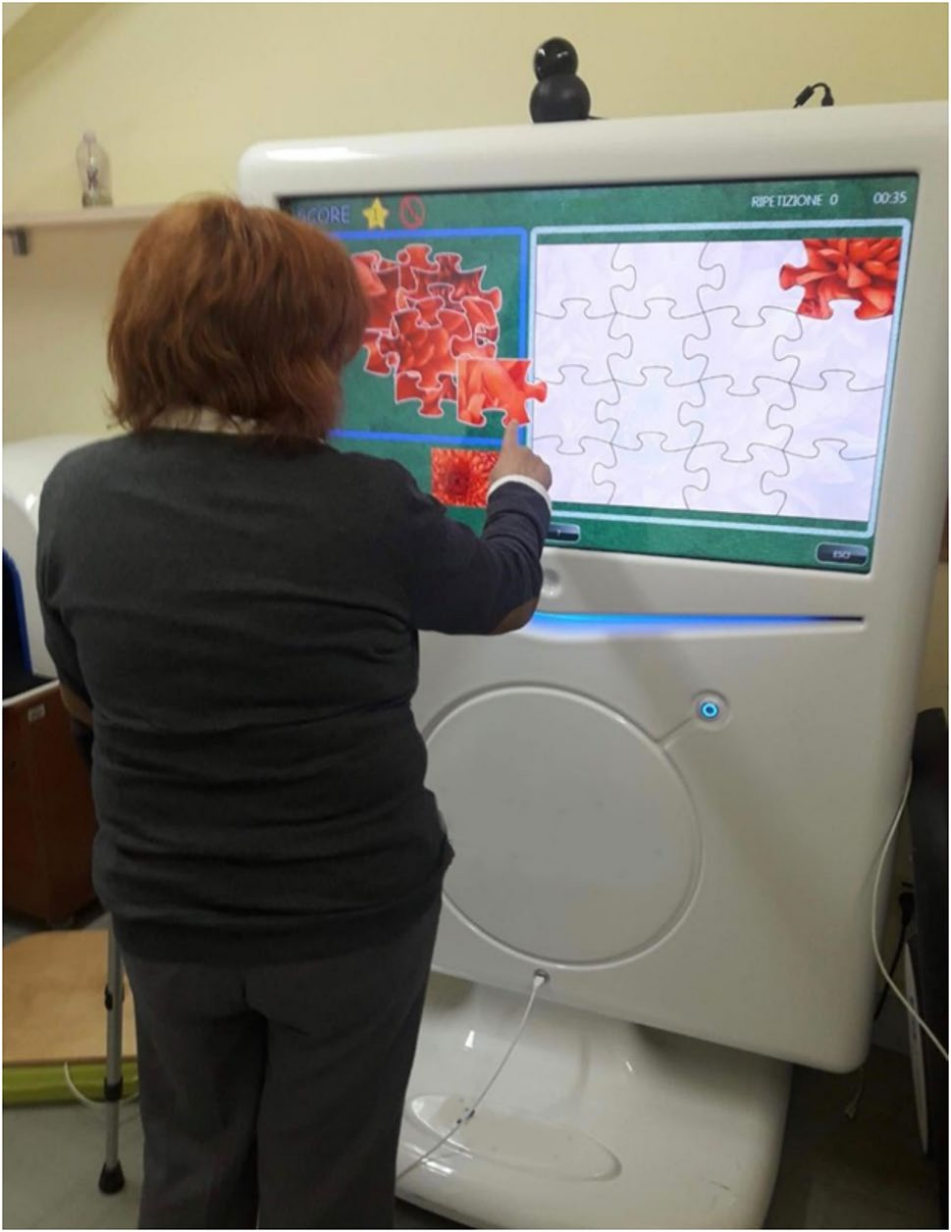
Patient while performing cognitive task with VRRS System.

**Fig. 2:**
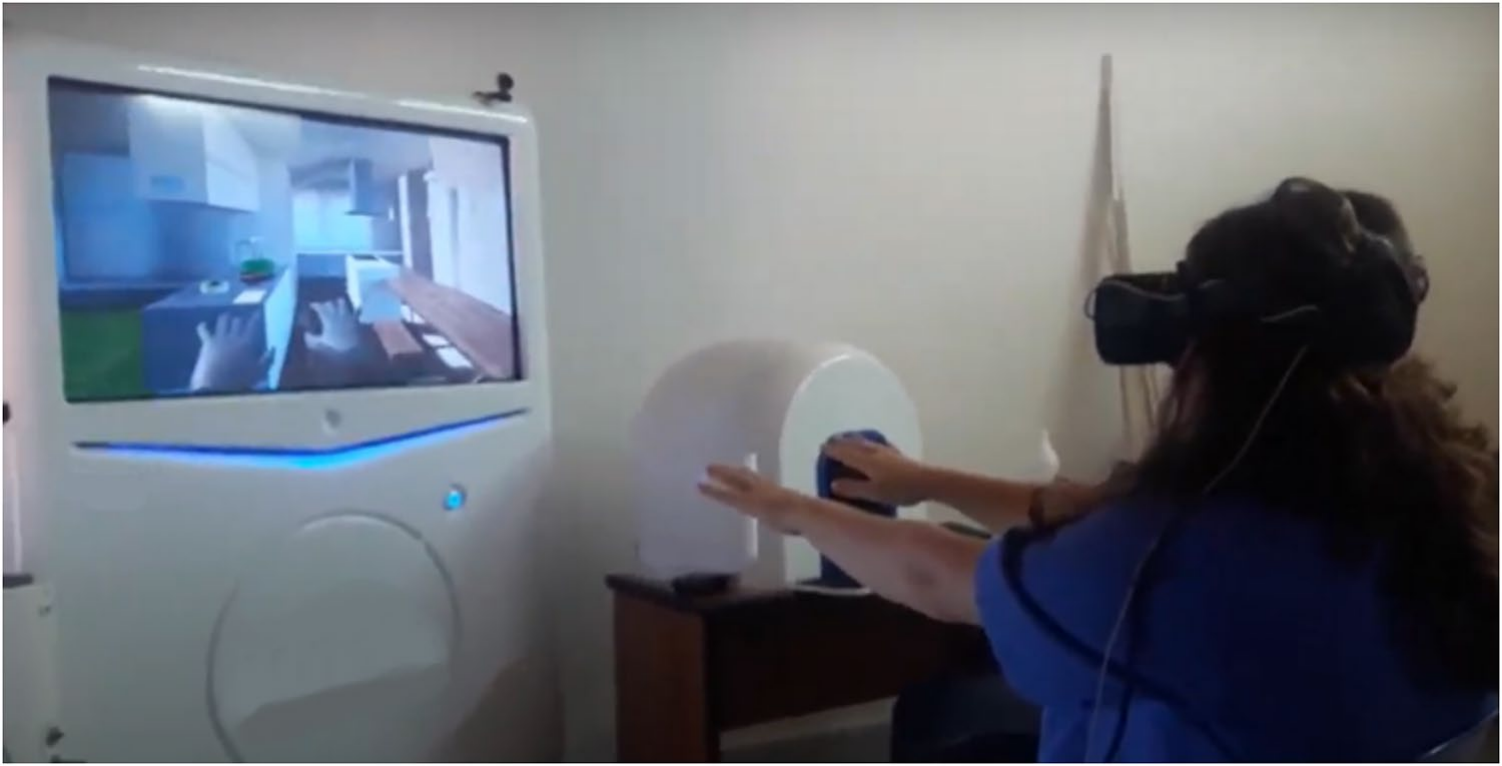
Patient while performing cognitive task with VRRS System. The patient interacts in a virtual kitchen environment by performing goal-directed movements (e.g., touching, manipulating objects), such as opening the fridge or cooking. Tasks involve ideomotor sequences of increasing complexity and provide immediate feedback, aiming to enhance executive functions.

For the EG, cognitive rehabilitation was conducted using the VRRS (Khymeia, Padua, Italy), an internationally patented Class I-certified medical device (Figure 1). The system provides augmented feedback during the exercises, enabling participants to receive immediate, real-time responses to their performance^42^. This interactive feedback not only enhances engagement but also helps users understand their progress and areas needing improvement. The VRRS training program involved both non-immersive 2D exercises, displayed on a screen (Figure 1), and immersive 3D exercises using a head-mounted display (Figure 2), depending on the task type and cognitive target. Specifically, 2D formats were employed for activities such as puzzle-solving and logical reasoning, where immersion was not essential, while the 3D modality was used for exercises requiring spatial navigation, embodiment, and sensorimotor integration. The level of immersion was thus modulated in a homogeneous and task-driven manner during each session. This choice allowed the rehabilitation to be adapted to the cognitive load and functional goals of each task. A supplementary table has been added (Table S1) to better clarified the training program.

All sessions were supervised by a trained therapist, who monitored performance, adjusted task difficulty in real time, and ensured personalized progression^43,44^. Tasks included reproducing visual sequences, solving logical problems, performing categorization, and managing dual-task demands. Difficulty was modulated using a three-tiered system (easy, intermediate, difficult), based on performance metrics such as accuracy and response time. In addition, the system’s internal monitoring functions logged adherence and performance data, while automatic reporting provided therapists with continuous feedback to adapt the rehabilitation plan as needed^45^.

For the Control group, cognitive rehabilitation was provided using conventional, paper-and-pencil methods based on standard neuropsychological training protocols. The intervention consisted of therapist-guided cognitive exercises targeting the same domains addressed in the experimental group, such as attention, memory, executive functions, and visuospatial abilities. Tasks included visual memory drills, verbal fluency exercises, logical reasoning activities, paper-based puzzles, and problem-solving tasks (Table 2). All sessions were conducted individually, in a quiet room and under the supervision of a trained neuropsychologist. Exercises were selected and adapted to the patient’s cognitive profile and performance level, ensuring progressive task difficulty and personalized engagement. Task complexity was modulated over time to maintain an appropriate challenge level and encourage cognitive improvement. The duration and frequency of sessions were matched to those of the experimental group. Therapists recorded patient adherence and progress manually using standardized tracking forms. Although this approach lacked the real-time feedback and multisensory stimulation provided by the VRRS, it represented the standard of care in cognitive rehabilitation and served as an appropriate comparator to assess the added value of VR-enhanced interventions.

### Statistical analysis

The Shapiro–Wilk test was conducted to assess the normality of the variables’ distribution. Continuous variables were expressed as mean ± standard deviation for normally distributed data and as median and interquartile range (first-third quartile) for non-normally distributed data, in accordance with the results of the normality test.. This choice aimed to provide a clear and accurate summary of the data. Student’s paired t-test or the Wilcoxon signed-rank test was used for within-group comparisons, while Student’s unpaired t-test or the Mann–Whitney U test or Chi-square test was applied for between-group comparisons, depending on the distribution of the data. A supplementary tables has been added (Table S2 and Table S3), where the type of statistical test applied (parametric or non-parametric) is explicitly indicated for each variable, along with the corresponding test statistic and effect size. Effect sizes were calculated using Cohen’s d for t-tests and effect size r for Wilcoxon tests and Mann–Whitney U tests. To assess whether changes over time were associated across domains, we calculated delta scores (T1–T0) for each variable and examined their relationships using Spearman’s rank correlation coefficient. This allowed us to investigate whether changes in motivational dimensions were related to concurrent changes in cognitive and emotional functioning. To assess the clinical relevance of the observed changes, we compared the individual pre-post differences with the Minimal Clinically Important Difference (MCID) reported in the literature. For the MoCA, a change of ≥ 2 points has been suggested as clinically meaningful in stroke populations^46^; for the HAM-D, a threshold of ≥ 3 points has been proposed^47^; and for the HAM-A, ≥ 2 points is commonly adopted^48^. Statistical analyses were performed using the open-source R software (version 4.2). The following R packages were used: stats, effsize, corrplot, ggcorrplot, ggplot2, reshape2, stringr, writexl, lm.beta, and QuantPsyc. A 95% confidence level was set, with an alpha error of 5%. Statistical significance was defined at p < 0.05.

## Results

A total of 54 patients were included in the study, with 27 participants in each group (experimental group and control group). No dropouts or adverse effects were reported during the intervention period. Baseline demographic and clinical characteristics of the two groups are reported in Table 3. No significant differences were found between groups in terms of gender distribution (χ^2^ = 0;df = 1;p = 1), age (p = 0.56), education level (p = 0.05), confirming that the two groups were comparable at baseline.

**Table 3:** Demographic characteristics of experimental and control groups.

	Experimental group	Control group	P-value
N. subjects	27	27	
Gender			
Male	17 (63%)	17 (63%)	1
Female	10 (37%)	10 (37%)	
Age years (Mean ± SD)	56.0 ± 8.55	53.8 ± 11.5	0.35
Education (Mean ± SD)	2.70 ± 0.67	5.0 ± 4.48	0.05

**Fig. 3:**
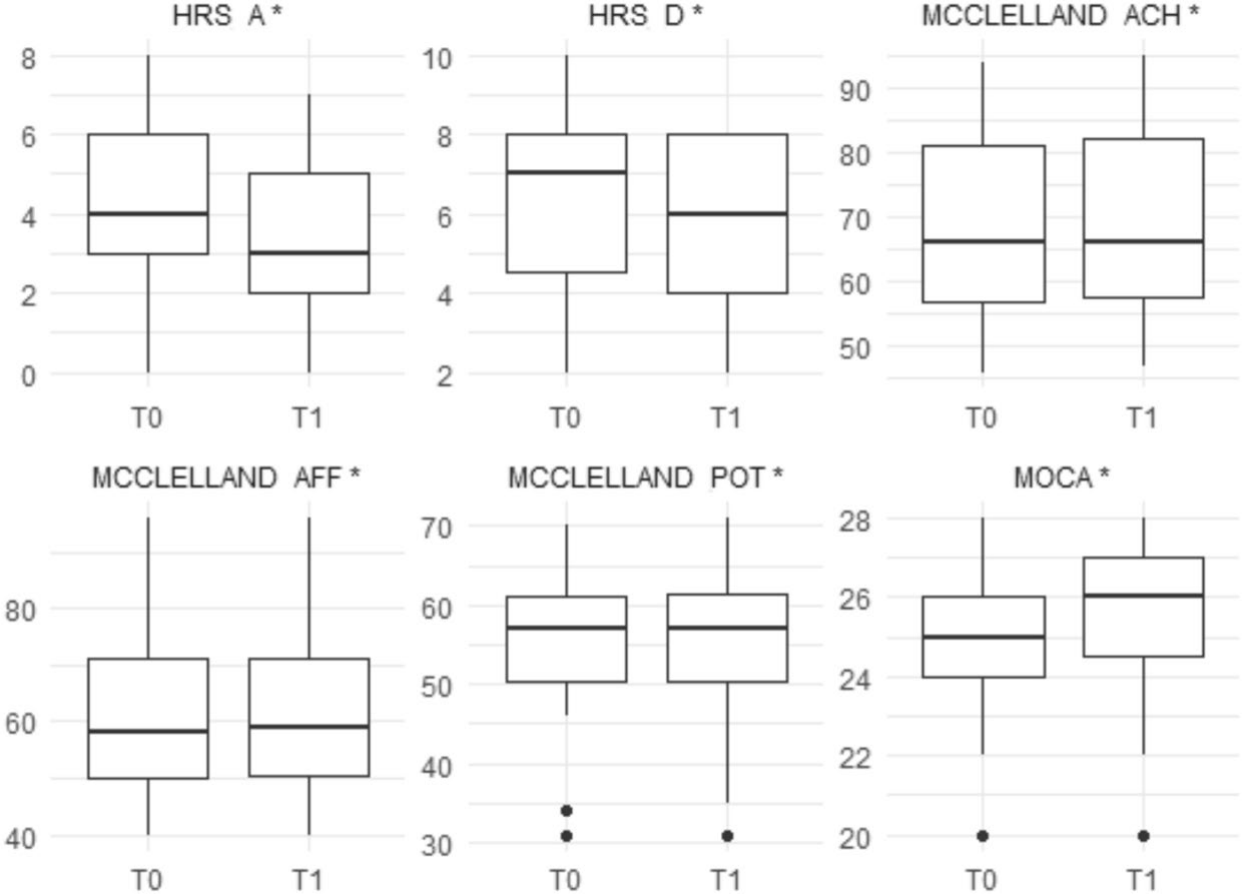
Boxplots of clinical scales in the VR group (pre- and post-intervention). Measurements are shown at T0 (pre) and T1 (post). Variables marked with * indicate a statistically significant difference between T0 and T1 (*p* < 0.05, Wilcoxon signed-rank test).

**Fig. 4:**
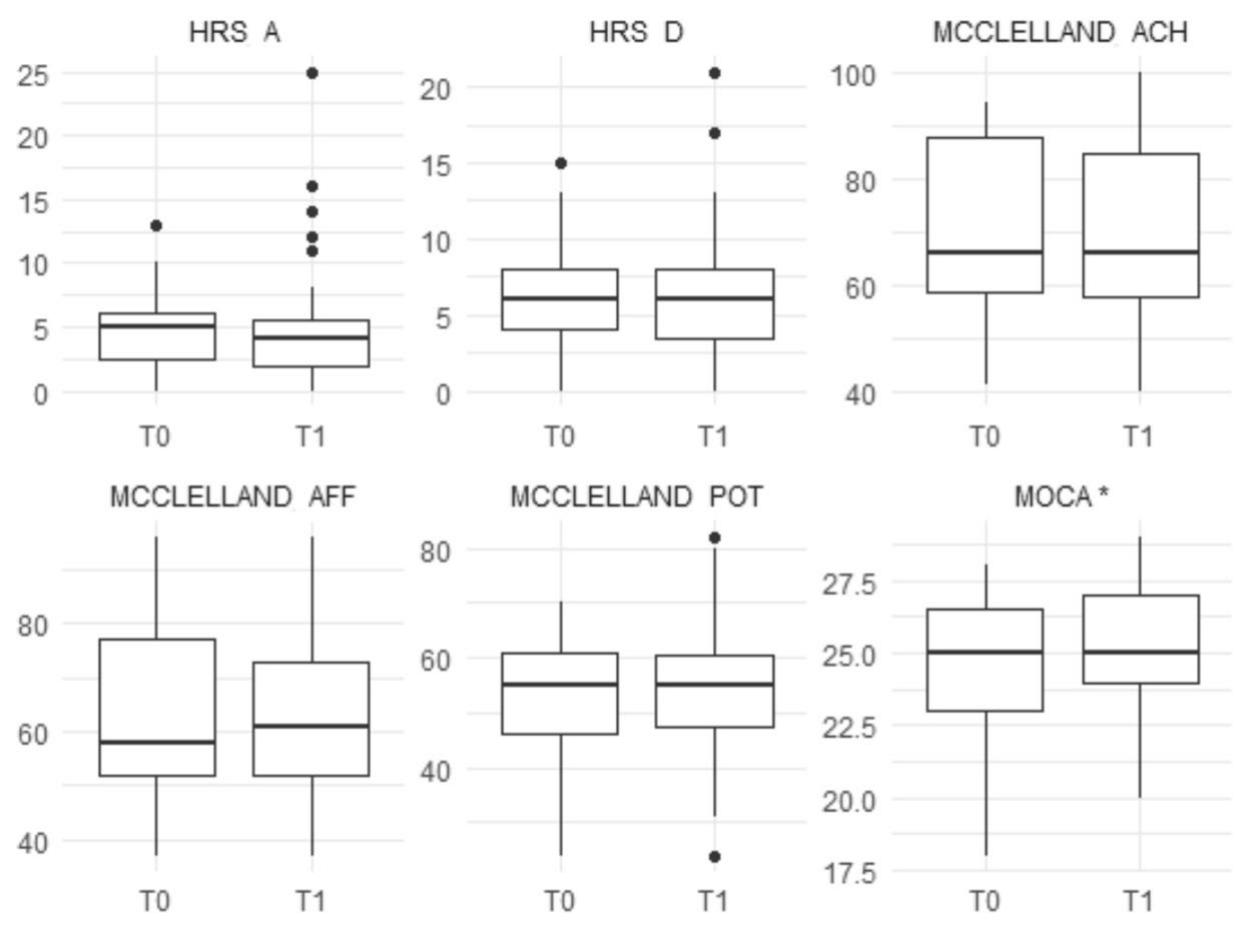
Boxplots of clinical scales in the HC group (pre- and post-intervention). Measurements are shown at T0 (pre) and T1 (post). Variables marked with * indicate a statistically significant difference between T0 and T1 (*p* < 0.05, Wilcoxon signed-rank test).

Intra-group analysis revealed significant differences, particularly in the experimental group (Table 4). Significant improvements were observed in MOCA (p = 0.001), HRS-D (p < 0.001), McClelland ACH (p < 0.001), McClelland AFF (p = 0.01), and McClelland POW (p = 0.0002) (Fig. 3). In contrast, the Control Group showed significant differences only in MOCA (p < 0.001) (Fig. 4). Between-group analysis revealed no significant differences between the two groups. Notably, for some comparisons, significant p-values were observed despite unchanged medians. This is due to the Wilcoxon signed-rank test’s sensitivity to consistent directional changes across individuals, which could not be reflected in summary measures such as the median.

**Table 4:** Socio-demographic characteristics and clinical scores of groups. Data are presented as medians (first and third quartiles) for non-normally distributed variables.

	Experimental group	Control Group	p-value
MOCA T0	25 (24–26)	25 (23–26.5)	1
MOCA T1	26 (24.5–27)	25 (24–27)	0.79
P-value	0.001*	< 0.001*	
HRS-D T0	7 (4.5–8)	6 (4–8)	0.77
HRS-D T1	6 (4–8)	6 (3.5–8)	0.97
P-value	0.003*	0.19	
HRS- A T0	4.26 ± 1.99	4.73 ± 2.78	0.63
HRS-A T1	3 (2–5)	4 (2–5.5)	0.31
P-value	< 0.001*	0.11	
McClelland ACH T0	66 (57–81)	66 (58.5–87.5)	0.59
McClelland ACH T1	66 (57.5–82)	66 (57.5–84.5)	0.97
P-value	< 0.001*	0.98	
McClelland AFF T0	58 (50–71)	58 (52–77)	0.51
McClelland AFF T1	59 (50.5–71)	61 (52–73)	0.56
P-value	0.01*	0.74	
McClelland POW T0	57 (50.5–61)	55 (46–61)	0.35
McClelland POW T1	57 (50.5–61.5)	55 (47.5–60.5)	0.4
P-value	0.0002*	0.16	

Montreal Cognitive Assessment (MoCA); Hamilton Depression Rating Scale (HAM-D); Hamilton Anxiety Rating Scale (HAM-A); McClelland Test – Achievement (ACH); McClelland Test – Affiliation (AFF); McClelland Test – Power (POW).

**Fig. 5:**
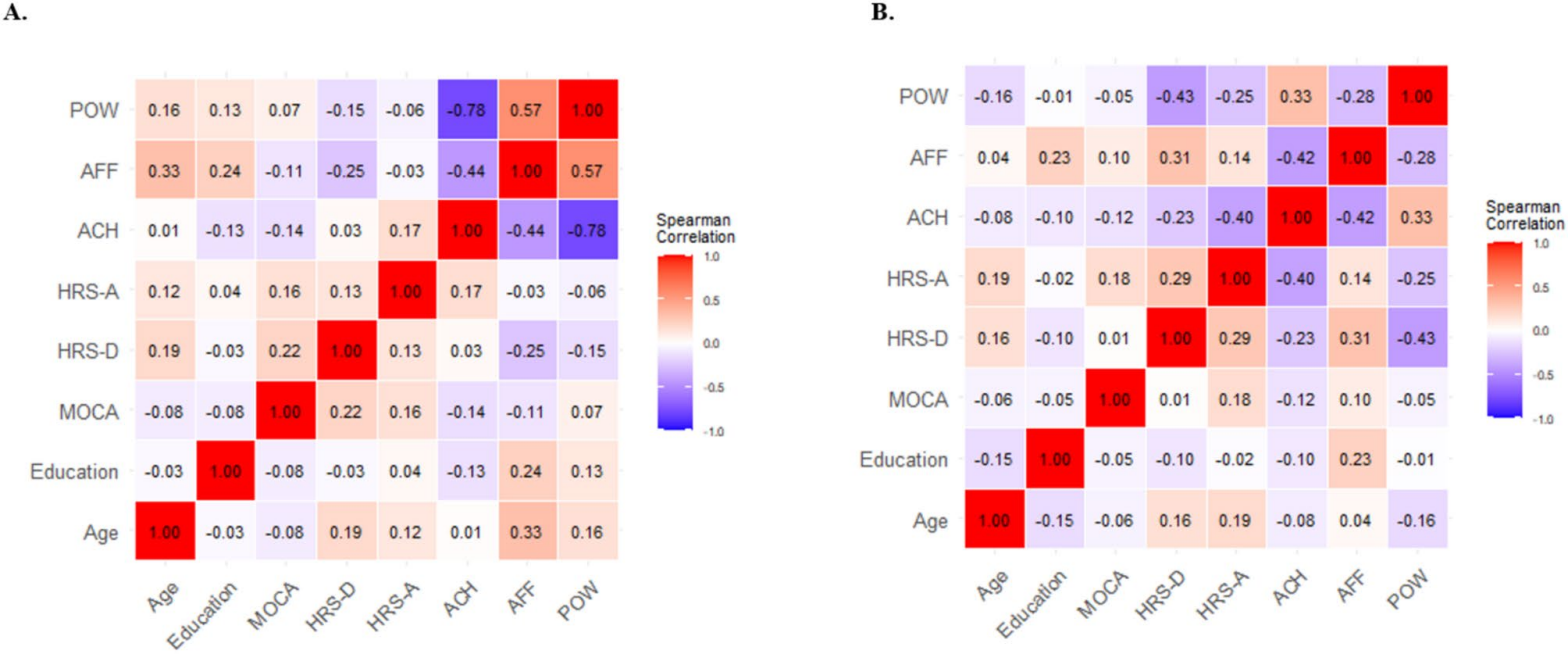
Correlation Matrices for Experimental Group and Control Group. **A**) The correlation matrix for the Experimental group; **B**) The correlation matrix for the control group. The size and color of the circles indicate the strength and direction of the correlations: red indicates positive correlations and blue indicates negative correlations. Legends: Montreal Cognitive Assessment (MoCA); Hamilton Depression Rating Scale (HAM-D); Hamilton Anxiety Rating Scale (HAM-A); McClelland Test – Achievement (ACH); McClelland Test – Affiliation (AFF); McClelland Test – Power (POW).

In the experimental group (Fig. 5A), Spearman’s correlation analysis identified several significant relationships. A significant negative correlation was found between McClelland ACH and McClelland AFF (r = -0.44, p = 0.02) and between McClelland ACH and McClelland POW (r = -0.78, p < 0.001). Conversely, McClelland AFF and McClelland POW demonstrated a significant positive correlation (r = 0.57, p = 0.002).

In the Control Group (Fig. 5B), significant positive correlations were observed between HRS-D and HRS-A (r = 0.29, p = 0.04) and between McClelland ACH and McClelland POW (r = 0.33, p = 0.02). Significant negative correlations were noted between HRS-D and McClelland POW (r = -0.43, p = 0.002) and between HRS-A and McClelland ACH (r = -0.4, p = 0.004). Additionally, a trend was identified between HRS-A and McClelland POW (r = -0.25, p = 0.07).

Finally, in our sample, 29.4% of participants showed a clinically relevant improvement on the MoCA, 15.7% on the HAM-D, and 43.1% on the HAM-A. These findings suggest that, beyond statistical significance, a notable proportion of participants experienced improvements exceeding the minimum threshold considered meaningful in clinical practice.

## Discussion

The present study aimed to evaluate the effects of VR rehabilitation on motivation, cognitive function, and emotional state in post-stroke patients compared to conventional cognitive rehabilitation. Indeed, the experimental group demonstrated significant improvements in cognitive function, as indicated by higher MoCA scores, as well as enhanced emotional state and motivation. Notably, there were substantial increases in Achievement (ACH, p < 0.001) and Affiliation (AFF, p = 0.006) motivation, while Power motivation (POW) showed a significant decline (p = 0.0002). In contrast, the Control Group exhibited significant improvements solely in cognitive function, highlighting the broader benefits of VR rehabilitation on multiple domains. Furthermore, when considering the clinical significance of the improvements, a considerable proportion of participants exceeded the established MCID thresholds, particularly for cognitive performance and anxiety. This reinforces the potential of the intervention not only to produce statistically detectable effects but also to yield meaningful changes in patients’ perceived functioning and emotional health.

The substantial improvements in cognitive performance observed in the experimental group highlight the transformative potential of immersive environments in promoting cognitive recovery following a stroke (Aderinto et al., 2023). VR provides dynamic, interactive, and highly customizable tasks that meaningfully engage patients, promoting sustained attention and encouraging neuroplasticity through real-time feedback mechanisms (Laver, 2020; Mani Bharathi, 2024). The VRRS system used in this study was specifically applied for cognitive rehabilitation and incorporated both non-immersive 2D exercises, displayed on a screen, and immersive 3D exercises, delivered through a head-mounted display. Depending on the cognitive domain targeted, exercises were selected to optimize engagement and cognitive load. The system provided an interactive visual interface and basic auditory feedback (e.g., verbal instructions and task-related sounds), but did not include proprioceptive or haptic inputs in the configuration used. The protocol included tasks targeting attention, memory, executive functions, and visuospatial abilities, which were personalized to each participant’s performance level^43,44^. Previous studies have reported that VR interventions, including cognitive training programs, could enhance attentional engagement and support functional recovery through enriched environments and interactive feedback^49,50^.

Although significant improvements in MoCA scores were also observed in the control group, this result appears less clinically relevant when compared to the experimental group. Specifically, the Control Group failed to show concurrent improvements in other domains, such as emotional state or motivation, highlighting the broader impact of VR beyond cognitive recovery. These cognitive improvements could reflect enhanced functional adaptation to rehabilitation stimuli, highlighting the brain’s intrinsic potential for recovery when exposed to innovative and personalized interventions^28,51^. Task-specific, repetitive, and contextually enriched environments, such as those offered by VR, are highly effective in promoting neuroplasticity, thereby maximizing learning and functional recovery in stroke rehabilitation (Darekar, 2023). These environments provide targeted stimulation, allowing patients to engage in goal-oriented tasks that mimic real-life scenarios^16,17^. Moreover, cognitive recovery plays a pivotal role in achieving functional independence and improving the overall quality of life for stroke survivors^7,52,53^. Cognitive deficits can significantly impair a survivor’s ability to perform essential tasks, maintain social relationships, and participate in meaningful activities, making their recovery critical for reintegration into society and achieving long-term well-being^52^. By combining the benefits of VR with evidence-based rehabilitation practices, there is a potential to address both physical and cognitive impairments, offering a comprehensive approach to stroke recovery. We observed a significant reduction in depression levels, as indicated by HRS-D scores within the experimental group. This effect was not observed for anxiety levels (HRS-A), indicating that the primary psychological benefit of VR rehabilitation lies in its impact on depressive symptoms. Prior research has demonstrated that immersive environments can alleviate psychological distress by providing safe and supportive virtual spaces that promote emotional regulation and engagement^53–55^. Interestingly, a significant negative correlation emerged in the Control Group between depression and Power motivation (as per McClelland POW). This suggests that reductions in depressive symptoms are associated with increased motivational scores, even in conventional rehabilitation settings. VR appears to actively modulate these interactions by integrating emotional and motivational support. VR may support emotional engagement by promoting a sense of autonomy and control^56^. Emotional state is critical for rehabilitation outcomes, influencing patient engagement, adherence to treatment, and long-term recovery trajectories^57^. While some associations between affective and motivational variables emerged, these findings are based on correlational data and should be interpreted with caution. The ability of VR to simultaneously address cognitive and emotional domains underscores its holistic approach to rehabilitation. Emotional state, often overlooked in traditional rehabilitation settings, is critical for sustaining motivation and compliance^17^. VR seamlessly combines cognitive tasks with emotionally supportive elements, such as soothing virtual environments, gamified progress tracking, and opportunities for social interaction. This integration makes VR a powerful tool not only for facilitating recovery but also for enhancing the overall patient experience and well-being^18^.

Another significant finding of our study concerns the effects of VR on motivation, as indicated by the McClelland test^41^. Among the motivational dimensions, ACH showed the most pronounced improvement in the VR group, while AFF increased moderately and POW significantly decreased. Although the McClelland test is based on a different theoretical tradition than SDT, we adopted SDT as an interpretive lens for understanding motivational changes. Specifically, the reduction in POW motivation and the concurrent increase in ACH may reflect a shift toward more autonomous, self-determined engagement, consistent with internalized motivation^58^. A strong negative correlation between ACH and POW (r = -0.78, p < 0.001) suggests that as participants became more focused on personal mastery, their drive for dominance or control diminished. This motivational reorientation may stem from the structure of VR training, which emphasizes individual progress, real-time feedback, and self-efficacy rather than external validation. The significant improvement in ACH underscores the importance of goal-oriented tasks, which promote sustained engagement, critical for successful rehabilitation. While gains in AFF were less marked, this dimension remains important, particularly for patients at risk of social isolation. Although prior studies suggest that VR environments with interactive or collaborative elements may help meet the need for relatedness^59^, our intervention did not include social features, nor did we assess social functioning directly. Therefore, any interpretation regarding social engagement should be made cautiously. Still, the observed trend in AFF may inform future designs that explicitly integrate social tasks to support relatedness. A moderate negative correlation was also observed between ACH and AFF (r = -0.44, p = 0.02), suggesting that a strong emphasis on achievement may, in some cases, reduce motivation related to social connection. This finding highlights the importance of balancing motivational dimensions: integrating therapist interactions or peer-based activities in VR could help sustain AFF without compromising ACH, fostering a more holistic recovery experience. Finally, while the correlation analyses offer meaningful insights into motivational dynamics, it is important to acknowledge their non-causal nature. The associations observed cannot be interpreted as evidence of direct psychological mechanisms. Nevertheless, these findings suggest that VR may support adaptive motivational profiles by reinforcing goal-directed behavior and internalized motivation while minimizing reliance on dominance-driven motives. The interplay between these motivational dimensions highlights the complexity of VR rehabilitation and its ability to target intrinsic drivers of behavior. By promoting intrinsic motivation, VR not only enhances task engagement and adherence but also supports a more adaptive and balanced motivational profile. These findings highlight the importance of designing VR interventions that leverage achievement-oriented tasks while integrating social components to optimize engagement and recovery outcomes. While virtual reality can include goal-oriented tasks and promote social interaction, it is critical that future interventions fully leverage these capabilities to improve engagement and psychosocial outcomes in post-stroke rehabilitation^60^.

### Implications for clinical practice

Our findings demonstrate that the benefits of VR in stroke rehabilitation are not limited to cognitive and emotional improvements but also significantly enhance patient motivation, a key element for the success of the rehabilitation process. Notably, even within the conventional rehabilitation group, significant correlations were found between emotional states and motivation, such as the negative relationship between depression levels (HRS-D) and Power motivation. This underscores that the interplay between emotional state and motivational dimensions is not unique to VR, but rather a core component of the rehabilitation process as a whole. Nonetheless, patients undergoing VR got better results, further highlighting the potential of this tool in the rehabilitation field.

These results highlight the importance of systematically monitoring the relationship between emotional states and motivation across various rehabilitation settings. By identifying individual emotional and motivational profiles, clinicians could tailor interventions more effectively, optimizing recovery outcomes and fostering greater patient engagement throughout the rehabilitation process. This personalized approach could enhance the overall effectiveness of treatment, addressing both the psychological and motivational aspects of recovery. Future studies should consider integrating functional cognitive assessments or real-world tasks to better capture the generalization of cognitive improvements to daily life performance.

VR environments foster intrinsic motivators, such as a sense of achievement and affiliation, offering engaging and goal-oriented activities that stimulate active participation. This increased motivation can translate into better treatment adherence and more lasting outcomes, increasing patients’ likelihood of being engaged in their rehabilitation programs^61^. However, the study data revealed a slight decrease in power-related motivation, indicating the need for a more thoughtful design of VR interventions. It is essential to strike an effective balance between autonomy and structure: while an overly structured environment could limit a sense of personal control, excessive autonomy might lack the necessary support to facilitate meaningful progress.

To address these challenges, personalized VR environments tailored to individual motivational profiles are essential. These environments would enable the design of rehabilitation experiences that align with each patient’s specific needs and preferences, maximizing engagement and effectiveness. Additionally, integrating adaptive features like dynamic task difficulty adjustments and targeted feedback systems can foster a balanced approach to motivation, ensuring sustained participation and significant progress in post-stroke rehabilitation.

### Strengths and limitations of the study

While this study provides promising insights, it has certain limitations that should be acknowledged. The reliance on self-reported measures introduces a potential for bias, as participants’ perceptions could not fully align with objective outcomes. Another limitation is the short follow-up period, which precludes a comprehensive understanding of the long-term effects of VR-based rehabilitation. Future research should address these limitations by employing larger, more diverse samples to provide a more representative analysis of VR’s effectiveness across various populations. Longitudinal study designs would be especially valuable for assessing the long-term impact and sustained outcomes of VR-based rehabilitation.

Notably, our protocol did not include neurophysiological measures (e.g., neuroimaging) to directly assess brain plasticity. Therefore, any reference to neural reorganization should be interpreted cautiously and as consistent with prior literature, rather than as a direct outcome of our intervention. The integration of objective measures, such as neuroimaging techniques or physiological markers, could provide a deeper understanding of the neurophysiological mechanisms driving the observed benefits of VR. This would not only enhance the validity of the findings but also inform the development of more targeted and effective rehabilitation protocols.

Furthermore, although statistically significant cognitive improvements were observed in the experimental group, only a limited number of participants exceeded the MDD threshold of 4 points on the MoCA. This could raise concerns about the robustness of the effect. However, it is important to note that in neurorehabilitation settings, especially among individuals with chronic stroke, such a magnitude of change is rarely observed. Clinically meaningful improvements are often more subtle and could still reflect enhanced engagement, attentional capacity, and executive functioning. For this reason, the MCID of approximately 2 points was considered a more appropriate reference for interpreting cognitive change. Future studies should continue to explore the clinical relevance of small but consistent improvements, possibly integrating real-life functional assessments alongside cognitive tests.

It is also important to note that our study did not aim to assess whether the level of immersivity (2D non-immersive vs. 3D immersive with head-mounted display) influenced participants’ outcomes. Given that both modalities were employed based on task demands within the same training sessions, the intervention was delivered in a functionally homogeneous way. However, the differential impact of immersion level on cognitive or emotional responses remains an open question. Future studies should specifically investigate this aspect, as it may provide further insight into how different VR configurations contribute to neurorehabilitation efficacy.

Furthermore , while immersive VR environments are designed to simulate real-life contexts and hold promise for enhancing functional abilities, our study did not directly assess whether the observed cognitive improvements translated into better performance in everyday activities. As a result, interpretations regarding the ecological validity and real-world transfer of these gains should be made with caution. The intervention was specifically focused on cognitive functions such as attention, memory, and executive functions, and the outcomes assessed were limited to neuropsychological and motivational measures. Future research would benefit from including functional or ecological outcome measures to better evaluate the practical applicability and broader impact of VR-based cognitive rehabilitation.

Lastly, our inclusion criteria excluded individuals with severe cognitive impairment (i.e., MoCA ≤ 18), as these patients may not be able to fully comprehend instructions or actively engage in VR-based interventions. While this was necessary to ensure adherence and safety, it limits the generalizability of our findings to individuals with milder cognitive deficits. Future research should focus on developing more accessible and adaptive VR systems that can be effectively used by individuals with severe cognitive impairments, thereby broadening the applicability of this rehabilitation approach. Additionally, although MoCA scores greater than 25 are typically considered within the normal range, subtle cognitive impairments may still be present and not fully captured by the scale. This ceiling effect may reduce the range for potential improvement in high-functioning participants, making small changes (e.g., from 26 to 27) less detectable or less clinically meaningful than larger improvements in participants with lower baseline scores. This limitation should be taken into account when interpreting pre-post differences using MoCA-based cut-off values for clinical significance.

Nevertheless, this study has several key strengths that enhance its contribution to the rehabilitation field. It takes an innovative approach by focusing on the use of VR rehabilitation to improve not only functional outcomes but also patient motivation, a crucial, yet often overlooked, factor in achieving long-term rehabilitation success. The use of well-established and validated assessment tools ensures the reliability of the collected data and provides a comprehensive assessment of key domains such as motivation, cognition, and emotional state.

Furthermore, the study’s focus on the motivational aspects of VR provides valuable insights into how such interventions can foster intrinsic motivation, enhancing patient adherence and engagement. Clinically, the findings offer practical implications for designing and implementing VR interventions, emphasizing the importance of tailoring experiences to individual patient profiles to optimize outcomes. Finally, the study highlights areas for improvement in VR activity design, setting a foundation for future research focused on developing more effective and personalized rehabilitation protocols.

## Conclusion

This preliminary RCT provides early evidence supporting the integration of VR into stroke rehabilitation, suggesting its potential to enhance cognitive function, alleviate emotional distress, and foster intrinsic motivation. The observed interactions between cognitive, emotional, and motivational domains underscore the holistic potential of VR as a multifaceted therapeutic tool. To fully harness this potential, future research should focus on refining VR protocols, exploring long-term outcomes, and expanding the applicability of VR across diverse populations. By ensuring that interventions are personalized to address the unique and complex needs of stroke survivors, we can optimize rehabilitation effectiveness and promote sustained recovery.

## Supplementary Information

Supplementary Information.

## Data Availability

The data supporting the findings of this study are available on request from the corresponding author. The data are not publicly available due to privacy or ethical restrictions.
