# Supplementary material for: The role of virtual reality-based cognitive training in enhancing motivation and cognitive functions in individuals with chronic stroke
Source: Sci Rep. 2025 Jul 12;15:25258. doi: 10.1038/s41598-025-08173-1 (PMC12255692; doi:10.1038/s41598-025-08173-1)
Supplement: Supplementary file 1 — Supplementary Information. [file 41598_2025_8173_MOESM1_ESM.docx]

**Table S1. Characteristics and mechanisms of the Virtual Reality Rehabilitation Systems (VRRS).**

| **Target Function/Skill Domain** | **How VRRS Addresses It** | **Mechanism of Action** | **Key Advantages** | **Limitations & Challenges** | **Example VR Scenarios/Tasks** | **Example System Types & Instrumentation** | **Clinical Considerations** | **Measurement & Outcome Tracking** |
| --- | --- | --- | --- | --- | --- | --- | --- | --- |
| **Attention & Processing Speed (Johansen et al., 2024).** | Presents engaging, dynamic tasks requiring sustained focus, selective filtering, and rapid responses. | Enhances cognitive functions through repetitive, targeted stimulation; increased motivation leads to longer engagement; provides immediate feedback on performance. | Highly motivating & engaging; customizable difficulty levels; objective performance data; controlled environment for managing distractions. | Potential for cybersickness (nausea, dizziness); transfer of skills to real-world contexts needs validation; hardware costs & space requirements. | Virtual driving/navigation requiring vigilance; reaction-time games; tasks demanding filtering of virtual distractors. | Type: Desktop VR, Tablet-based systems, sometimes non-immersive screen setups. Instrumentation: Standard PC/Tablet, Mouse, Keyboard, Touchscreen, basic Game Controllers. HMDs less common but possible for higher immersion. | Monitor patient tolerance (cybersickness); adjust cognitive load appropriately; integrate with therapist guidance for strategy training. | Reaction times; accuracy rates; error counts; task completion time; sustained attention duration within VR. |
| **Memory (Working, Episodic, Spatial) (Johansen et al., 2024).** | Creates immersive environments for encoding and retrieving information (sequences, locations, lists). | Leverages multi-sensory encoding; utilizes spatial navigation to engage hippocampal networks; gamified repetition aids consolidation. | Provides rich contextual cues for recall; allows practice of memory strategies in simulated real-life contexts (e.g., cooking or shopping); high engagement. | R tasks may not fully mimic real-world memory demands; risk of cognitive overload if tasks are too complex. | Virtual supermarket task (remembering a shopping list); kitchen, navigating a virtual town to find locations; sequence recall games | Type: Tablet Apps, Desktop VR, HMD-based systems (especially for spatial tasks). Instrumentation: Tablet/PC, Controllers, Touchscreen. HMD & Controllers for immersive spatial navigation. | Match task complexity to patient's cognitive capacity; ensure tasks are meaningful and relatable; manage potential frustration. | Recall accuracy (items, sequence, location); navigation efficiency (time, errors); learning rate across sessions. |
| **Executive Functions (Planning, Problem-Solving) (Shen et al., 2020)** | Simulates complex activities requiring planning steps, sequencing actions, adapting to unexpected events, and making decisions. | Offers a safe space to practice complex cognitive processes; allows for trial-and-error learning with immediate feedback; promotes development of problem-solving strategies. | Safe environment to make mistakes without real-world consequences; potential for simulating Activities of Daily Living (ADLs) realistically (ecological validity); adaptable complexity. | Difficulty in perfectly replicating real-world nuances and unpredictability; development of sufficiently complex and adaptive scenarios can be challenging. | Virtual cooking/kitchen tasks (sequencing steps, managing multiple items); simulated vocational tasks; planning routes in a complex virtual environment. | Type: Desktop VR or HMD-based systems offering interaction with virtual objects/environments. Instrumentation: PC, HMD, Hand Controllers, potentially Hand Tracking sensors (e.g., Leap Motion). | Start with structured tasks and gradually increase complexity/autonomy; provide therapist feedback on planning strategies; debriefing after sessions is crucial. | Task completion time/efficiency; number of steps correctly planned/sequenced; error analysis; strategy effectiveness ratings. |
| **Visuospatial Skills & Perception (Pastel, 2022)** | Utilizes 3D immersive environments requiring spatial navigation, object localization/manipulation, depth perception, and visual field scanning. | Provides strong visual and vestibular input; forces attention across the visual field (useful for neglect); facilitates spatial mapping and body schema awareness. | Realistic simulation of 3D space; objective tracking of gaze and head movements possible; specific protocols can target visual field deficits (e.g., neglect). | Potential for visual strain or disorientation; hardware limitations (field of view, resolution) can impact realism; may exacerbate existing vestibular issues. | Virtual maze navigation; object finding/sorting tasks in a 3D space; reaching and grasping virtual objects; prism adaptation tasks presented virtually for neglect. | Type: Primarily HMD-based systems for full immersion; Large Screen Projectors/CAVE systems are alternatives. Instrumentation: HMD, PC, Hand Controllers. Optional: Eye-tracking modules within HMDs. | Pre-screen for severe visual or vestibular impairments; monitor closely for dizziness or discomfort; ensure proper calibration for the user's interpupillary distance (IPD). | Navigation accuracy/time; search time/hit rate; range of visual field explored (eye/head tracking data); accuracy of spatial judgments. |

*Legend: Virtual Reality Rehabilitation System (VRRS), Attention & Processing Speed (APS), Reaction Time (RT), Working Memory (WM), Episodic Memory (EM), Spatial Memory (SM), Executive Functions (EF), Activities of Daily Living (ADLs), Visuospatial Skills & Perception (VSP), Three-Dimensional (3D), Head-Mounted Display (HMD), Eye-Tracking (ET), Motor Control (MC), Upper Limb (UL), Lower Limb (LL), Inertial Measurement Unit (IMU), Virtual Reality Gloves (VR Gloves), Range of Motion (ROM), Balance & Gait (BG), Center of Pressure (COP), Instrumented Treadmill (IT), Motion Capture System (MCS), Sensorimotor Integration & Coordination (SIC).*

**Table S2.** **Between-group comparisons (Experimental Group vs Control Group).** Test statistics, p-values, and effect sizes are reported for each variable. Wilcoxon rank-sum tests or independent-sample t-tests were used depending on the distribution.

|  | **Test** | **Statistic** | **p-value** | **Effect Size** |
| --- | --- | --- | --- | --- |
| MOCA T0 | Wilcoxon | U=688.5 | 1 | r=NA |
| MOCA T1 | Wilcoxon | U=663.5 | 0.79 | r=0.09 |
| HRS-D T0 | Wilcoxon | U=717 | 0.77 | r=0.08 |
| HRS-D T1 | Wilcoxon | U=692 | 0.97 | r=0.22 |
| HRS- A T0 | Wilcoxon | U=642.5 | 0.63 | r=0.04 |
| HRS-A T1 | Wilcoxon | U=593 | 0.31 | r=0.05 |
| McClelland ACH T0 | Wilcoxon | U=637.5 | 0.59 | r=0.03 |
| McClelland ACH T1 | Wilcoxon | U=685 | 0.97 | r=0.22 |
| McClelland AFF T0 | Wilcoxon | U=626 | 0.51 | r=0.004 |
| McClelland AFF T1 | Wilcoxon | U=633 | 0.56 | r=0.02 |
| McClelland POW T0 | Wilcoxon | U=778 | 0.35 | r=0.04 |
| McClelland POW T1 | Wilcoxon | U=769.5 | 0.4 | r=0.03 |

Effect sizes are reported as r, calculated for non-parametric comparisons (Wilcoxon tests). The r effect size was calculated as $r=\frac{Z}{\sqrt{N}}$, where $Z$ is the standardized test statistic and $N$ is the total number of observations.

**Table S3.** **Within-group comparisons (pre- vs post-intervention) for the VR Experimental and HC Control groups.** Test statistics, p-values, and effect sizes are reported for each variable. Depending on the distribution of the data, Wilcoxon signed-rank tests or paired-sample t-tests were used.

|  |  | **Test** | **Statistic** | **p-value** | **Effect Size** |
| --- | --- | --- | --- | --- | --- |
| **Experimental Group** | MOCA | Wilcoxon | W=7 | 0.0012 | r=0.58 |
|  | HRS-D | Wilcoxon | W=66 | 0.003 | r=0.53 |
|  | HRS-A | Wilcoxon | W=231 | <0.001 | r=0.78 |
|  | McClelland ACH | Wilcoxon | W=0 | <0.001 | r=0.68 |
|  | McClelland AFF | Wilcoxon | W=0 | 0.01 | r=0.44 |
|  | McClelland POT | Wilcoxon | W=0 | 0.0002 | r=0.68 |
| **Control Group** | MOCA | Wilcoxon | W=61 | 0.0002 | r=0.50 |
|  | HRS-D | Wilcoxon | W=373.5 | 0.19 | r=0.12 |
|  | HRS-A | Wilcoxon | W=528 | 0.11 | r=0.17 |
|  | McClelland ACH | Wilcoxon | W=249.5 | 0.98 | r=0.30 |
|  | McClelland AFF | Wilcoxon | W=203 | 0.74 | r=0.09 |
|  | McClelland POT | Wilcoxon | W=189.5 | 0.16 | r=0.14 |

Effect sizes are reported as r, calculated for non-parametric comparisons (Wilcoxon tests). The r effect size was calculated as $r=\frac{Z}{\sqrt{N}}$, where $Z$ is the standardized test statistic and $N$ is the total number of observations.
